# Fowlpox virus recombinants expressing HPV-16 E6 and E7 oncogenes for the therapy of cervical carcinoma elicit humoral and cell-mediated responses in rabbits

**DOI:** 10.1186/1479-5876-8-40

**Published:** 2010-04-21

**Authors:** Antonia Radaelli, Eleana Pozzi, Sole Pacchioni, Carlo Zanotto, Carlo De Giuli Morghen

**Affiliations:** 1 Department of Medical Pharmacology, Università di Milano, Milan, Italy; 2 Department of Pharmacological Sciences, Università di Milano, Milan, Italy; 3 CNR Institute of Neurosciences, Cellular and Molecular Pharmacology Section, Università di Milano, Milan, Italy

## Abstract

**Background:**

Around half million new cases of cervical cancer arise each year, making the development of an effective therapeutic vaccine against HPV a high priority. As the E6 and E7 oncoproteins are expressed in all HPV-16 tumour cells, vaccines expressing these proteins might clear an already established tumour and support the treatment of HPV-related precancerous lesions.

**Methods:**

Three different immunisation regimens were tested in a pre-clinical trial in rabbits to evaluate the humoral and cell-mediated responses of a putative HPV-16 vaccine. Fowlpoxvirus (FP) recombinants separately expressing the HPV-16 E6 (FP_E6_) and E7 (FP_E7_) transgenes were used for priming, followed by E7 protein boosting.

**Results:**

All of the protocols were effective in eliciting a high antibody response. This was also confirmed by interleukin-4 production, which increased after simultaneous priming with both FP_E6 _and FP_E7 _and after E7 protein boost. A cell-mediated immune response was also detected in most of the animals.

**Conclusion:**

These results establish a preliminary profile for the therapy with the combined use of avipox recombinants, which may represent safer immunogens than vaccinia-based vectors in immuno-compromised individuals, as they express the transgenes in most mammalian cells in the absence of a productive replication.

## Background

Infection by human papilloma viruses (HPVs) represents the second most-common cause of malignancies in women worldwide, and the oncogenic activity of the E6 and E7 early proteins expressed by the high-risk HPV-16 mucosal genotype accounts for the majority of anogenital tumours [1]. E6 and E7 interfere with the host cell-cycle regulatory proteins p53 and p105Rb, leading to transformation and carcinogenesis [2], facilitate cell immortalisation in primary human keratinocytes [3], increase genomic instability [4], and maintain the transformed phenotype [5] of cervical cancer cells [6].

Prophylactic vaccines are the best choice of intervention against HPV, as they can inhibit infection and prevent clinical disease by neutralising the incoming virus. On this basis, capsid-L1-based virus-like-particles (VLPs) have been successfully used to elicit HPV-11 neutralising antibodies in a nude-mouse xenograft system [7], and the recombinant L1/L2 proteins were able to prevent infection [8]. In particular, VLPs have proven to be successful as prophylactic bivalent (Cervarix^®^, GSK) [9] and quadrivalent (Gardasil^®^, Merck) [10] HPV vaccines in women, by eliciting the production of virus-neutralising antibodies. More recently, a recombinant adenovirus carrying the HPV-16 L1 gene was shown to elicit complete protection in Rhesus macaques [11]. However, the long delay in tumour development after infection limits the assessment of the vaccine efficacy [12] and suggests the need to support the treatment of HPV-related precancerous lesions and tumours. Although extensive screening for early diagnosis has lead to a reduction in the mortality of women in the developed countries, there are around 500,000 new cases of cervical cancer each year which make the development of an effective therapeutic vaccine highly desirable.

As they are expressed throughout the replicative cycle of the virus, E6 and E7 might provide a target for therapeutic vaccines to clear an already established tumour. They have been therefore evaluated in preclinical studies for prophylaxis or therapy performing the challenge with transformed cells after or before vaccination [13]. Immunotherapy with E6 and E7, either alone or expressed as L2/E6/E7 fusion-protein by genetic DNA vaccines, showed tumour growth control [14-16] and induced HPV-specific cytotoxic T-lymphocytes (CTLs) targeted to cancer cells [17-20]. However, peptides and purified proteins, processed through the MHC class II, direct the immune response towards the Th2 phenotype and generally fail to induce an adequate level of CD8+ T-cells and a strong T-helper [21] response, so that a poor clinical efficacy is often obtained [21].

Vaccinia virus (VV) recombinants expressing the HPV-16 and HPV-18 E6 and E7 proteins have already been used in several clinical studies for the immunotherapy of cervical cancer [22-26]. Although many attempts were performed also with VV attenuated strains, such as the Modified Vaccinia Virus Ankara (MVA) [27,28], the replication of these viruses is only partially abortive [29], and safety concerns were raised due to the severe side effects of the vector in immuno-compromised subjects [30]. Avipox viruses have been developed as novel vaccines against human infectious diseases, as they are restricted for replication to avian species [31], permissive for entry and transgene expression in most mammalian cells, and immunologically non cross-reactive with vaccinia. They might represent therefore safer immunogens [32] which have never been used as vectors for HPV and can be administered to previously smallpox-experienced human beings.

Due to papillomavirus species specificity, no natural animal model is at present available to test human HPV vaccines. The immune response in rodents inoculated with E6- and E7-transfected cell lines has suggested their use to test the immunotherapy of HPV-related tumours [22]. Preclinical studies were successful in eliciting an immune response in the bovine [33], canine [34], murine, and cottontail rabbit papillomavirus (CRPV) models. In particular, CRPV produces transient or progressive skin warts in domestic rabbits, which can represent a simple animal model both for prophylaxis and therapy [35-38], when challenged with VX2T tumour rabbit cells [39].

In the present study, two new fowlpox recombinants expressing the HPV-16 E6 and E7 oncogenes (FP_E6 _and FP_E7_) were evaluated for the ability to elicit a complete immune response and protection in rabbits following prime-boost protocols where the two constructs were given either alone or in combination. In these animals, we also found that it is possible to evaluate a CTL response by using syngeneic Ag-specific SV40-immortalized target cells, and either expanded CTLs or fresh peripheral blood mononuclear cells (PBMCs) as effector cells.

## Methods

### Cells

Specific-pathogen-free primary chick embryo fibroblasts (CEFs) were grown in Dulbecco's Modified Eagle's Medium (DMEM) supplemented with 5% heat-inactivated calf serum (CS; Gibco Life Technologies, Grand Island, NY, USA), 5% Tryptose Phosphate Broth (Difco Laboratories, Detroit, MI, USA), 100 U/ml penicillin and 100 mg/ml streptomycin (P/S). CaSki cells, containing multiple copies of integrated HPV-16 DNA, and green monkey kidney (Vero) cells were grown in DMEM supplemented with 10% CS and P/S. Rabbit PBMCs were obtained from heparinised rabbit blood and used for CTLs and cytokine assays; the PBMCs were grown in RPMI supplemented with glutamine, 10% FCS, and P/S. Rabbit skin fibroblasts were obtained with a 3-mm biopsy punch and immortalised with SV40 [40]; these were first grown in DMEM supplemented with 10% CS, 5% FCS, and P/S, and then they were used either with 2% (DMEM2) or 10% (DMEM10) FCS in DMEM. Rabbit VX2T cells, containing the complete CRPV genome [39], kindly supplied by Dr. F. Breitburd (Pasteur Institute, Paris, France), were grown on collagene-plated type 1 flasks (Iwaki, Scitech Division, Asahi Techno Glass, Tokyo, Japan) in DMEM supplemented with P/S, 5 μg/ml amphotericin B (Sigma-Aldrich, St. Louis, MO), 40 μg/ml gentamicin (Sigma), 6.5 ng/ml Epidermal Growth Factor (EGF) (Sigma), 0.5 μg/ml hydrocortisone (Sigma) and 2 mM L-glutamine (Sigma).

### Viruses

The FP_E6 _and FP_E7 _viruses were obtained by *in vitro *homologous recombination [41], amplified on CEFs, sucrose gradient purified, titred and used for animal immunisation. The FP recombinant containing the *env *gene of HIV-1 (FP*env*) [42] was used as an irrelevant negative control in the CTL assay.

### VX2T cells expansion and challenge with the minimal tumorigenic dose (MTD)

CD-1 nude mice (Charles River Lab., Calco, Italy), housed and handled in sterile condition, were inoculated subcutaneously in the leg with 1 × 10^7 ^VX2T cells. When the tumour reached around 1 cm^3 ^volume (1 month), the animals were sacrificed, and the carcinomas explanted. Tumour cells were minced in calcium- and magnesium-free phosphate-buffered saline (PBS^-^) pH 7.2, propagated again in CD-1 mice for a few cycles, until they were expanded on collagen-coated flasks, stocked and used to test the MTD for rabbit challenge. For the MTD test, two rabbits were inoculated intradermally (i.d.) on the upper back with a decreasing number of VX2T cells, starting from 1.8 × 10^7 ^in 200 μl of PBS^-^. The MTD dose able to generate a tumour in 6 days (10^7 ^cells) was used in 200 μl volume to challenge all of the animals by a single intradermal injection.

The presence of E6 and E7 genes in VX2T cells was assessed using CRPV primers V234 (5'-CTG-AGA-TCG-CAA-CGC-ATT-GC-3') and V235 (5'-GCC-TGG-ATA-TAA-TCC-AAG-TT-3') for E6 and V236 (5'-TAT-TTC-TGC-TAT-CCT-GTG-CG-3') and V237 (5'-GCC-ATT-TTC-AGT-TAC-AAC-AC-3') for E7. Amplifications were carried out starting from 30 ng of DNA in a final volume of 20 μl, in a mixture containing 1 μM of each primer, 200 μM of each dNTP, 2.5 μM MgCl_2_, 0.025 U/μl of Taq DNA polymerase (Fermentas, MMedical, Milan). PCR conditions were 95°C for 1 min followed by 30 cycles at 95°C for 45 sec, 55°C for 30 sec, 72°C for 1 min, and 72°C for 7 min in the PTC-200 thermocycler (MJ Research, Waltham, MA).

### Production of the HPV-16 E6 and E7 proteins

Expression plasmids pQE30 (Qiagen, Valencia, CA, USA) engineered to contain the E6 or E7 genes of HPV-16 [43] were kindly supplied by Dr. Giorgi (Istituto Superiore di Sanità, ISS, Rome, Italy), and called pQE30-E6/His and pQE30-E7/His. After cloning into JM109 bacterial cells, these were used for the production of the RGS His (H_6_) E6 and E7 tagged proteins as per manufacturer instructions (Qiagen), with minor modifications, and referred to as pE6 and pE7. Briefly, JM109/pQE30-E6/His bacterial cells were lysed in Phosphate Lysis Buffer (PLB, 300 mM NaCl, 1% Triton X-100, pH 8) in buffer A (10 mM Tris, 100 mM Na_2_HPO_4_, 6 M guanidine-HCl, pH 8). For JM109/pQE30-E7/His, cell lysis was in PLB in buffer B (10 mM Tris, 100 mM Na_2_HPO_4_, 8 M Urea, pH 8). After clarification for 30 min at 17,000 × g at 4°C, the supernatants of the E6 and E7 preparations were supplemented with 1% Triton X-100/20 mM imidazole pH 8 in buffer A or B, respectively, before incubating with Ni-NTA agarose resin (Qiagen) for 30 min at room temperature. After washing once with 1% Triton X-100 in buffer A or B, respectively, twice in buffer A or B, respectively, and multiple times with buffer C (100 mM Na_2_HPO_4_, 10 mM Tris, 8 M Urea, pH 6.3) to a final OD_280 _of 0.013, the proteins were eluted into different fractions with 1 M imidazole, pH 8. After separation by 15% SDS-PAGE, the fractions enriched in the recombinant proteins were pooled, quantified and stored at -80°C until use. The proteins were used both for the immunisation and in the ELISA assays. pE7 was dialysed overnight at 4°C using slide-A-lyser cassettes (10 kDa MW cut-off, Pierce, Rockford, IL) soaked in dialysis buffer (25 mM Tris-HCl, 100 mM NaCl).

### Immunisation protocols

**Figure 1:**
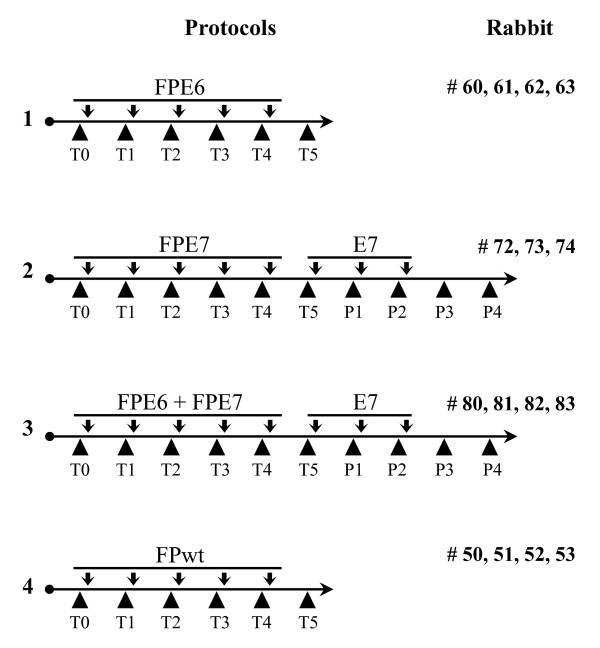
**Prime-boost protocols for rabbit immunisation**. Animals were immunised i.d. every month and bled before each inoculation. Four immunisation protocols were applied. In Protocol 1, the animals were immunised with the FP_E6 _recombinant (10^8 ^PFU/animal), in Protocol 2 with the FP_E7 _recombinant (10^8 ^PFU/animal), in Protocol 3 with the FP_E6 _+ FP_E7 _recombinants (10^8 ^PFU/recombinant/animal), in Protocol 4 with FPwt (10^8 ^PFU/animal). In Protocols 2 and 3 the animals were also boosted with the recombinant E7 protein (100 μg/boost). Rabbits # 50, 62 and 83 died for natural reasons before the fifth priming. T1-T5, immunisation times for priming; P1-P4, immunisation times for protein boost.

Four groups of two-month-old male New Zealand White rabbits (Charles River) were inoculated with multiple intradermal injections. Priming with the recombinant viruses was performed five times, at 3-4-week intervals (Fig. 1), with either FP_E6 _(Protocol 1, rabbits # 60, 61, 62, 63; 10^8 ^PFU/animal), or FP_E7 _(Protocol 2, rabbits # 72, 73, 74; 10^8 ^PFU/animal) or FP_E6 _plus FP_E7 _(Protocol 3, rabbits # 80, 81, 82, 83; 10^8 ^PFU/each recombinant/animal) or FPwt (Protocol 4, rabbits # 50, 51, 52, 53; 10^8 ^PFU/animal). The animals of Protocols 2 and 3 were also boosted three times with the recombinant E7 protein (100 μg/boost). Protein immunisations were performed in 50% v/v Freund's incomplete adjuvant. All of the rabbits remained in good health after all rounds of the immunisations. Rabbits # 50, 62 and 83 died before the fifth priming for natural reasons and do not appear in all of the tests. Bleedings were performed before each immunisation, from the ear central artery using heparin (200 μl), and are referred to as T1-T5 after priming immunisations, and as P1-P4 after the protein boosting. The plasma fractions were aliquoted and frozen at -80°C, and the PBMCs were used for the RNA extraction and CTL assays.

All the animals were housed and handled in accordance with the European guidelines no. 86/609/CEE and 116/92 for the protection of laboratory experimental animals and laboratory animal care (Ministry of Health, Department for Veterinary Public Health, Nutrition and Food Security, Protocol 17/2006).

### ELISA

The rabbit sera were immuno-adsorbed overnight at 4°C with FPwt-infected Vero cells and tested for the presence of antibodies against the HPV-16 E6 and E7 proteins before the first and after each immunisation. The ELISA was essentially performed as previously described [42]. Briefly, 96-well maxisorp microtitre plates (Nunc, Naperville, IL, USA) were coated with either pE6 (250 ng/well) in PBS^- ^or pE7 (25 ng/well) in 0.05 M carbonate-bicarbonate buffer, pH 9.6, and incubated overnight at 4°C. When CaSki lysates were used as a plate-bound immunogen (10^5 ^cells/well in the coating buffer used for pE7), after overnight incubation, the wells were masked with the 1:1000-diluted AbE7/Gi or AbE6/Gi antibodies (kindly supplied by Dr. Giorgi) for the E6 or E7 antibody determination, respectively. A preliminary test was also performed to find the appropriate serum dilution able to saturate alternatively one of the two CaSki antigens, and determine the relative contribution of each immunogen. The sera of the E6- and E7-immunised rabbits were then added at 1:25 or 1:250 dilutions, when proteins E6 or E7 were coated, or at 1:4000 dilution, when CaSki lysates were plated.

The binding was revealed by a 1:1000 dilution of goat anti-rabbit or goat anti-human horseradish-peroxidase-conjugated sera (Dako-Cytomation, Glostrup, Denmark) and tetramethylbenzidine (TMB) substrate (Sigma). The pre-immune rabbit serum for each animal was used as a negative control. The absorbance of each well was measured at 450 nm with a 550 Microplate Reader (Bio-Rad Lab., Hercules, CA, USA).

### RNA isolation and cytokine quantification

RNA extraction from PBMCs was performed at different times post-immunisation by Trizol LS (Gibco), as per manufacturer instructions. The RNAs from all of the samples were treated with 10 U RNase-free DNase I (Roche Diagnostics, Indianapolis, IN, USA) for 4 h at 37°C to eliminate any cellular or viral DNA. The RNA was then precipitated with 100% EtOH in the presence of 100 mM Na acetate, washed in 75% EtOH, and resuspended in diethylpyrocarbonate-treated water. Aliquots of 100 ng (in duplicate) were used to reveal the levels of expression of rabbit interferon (IFN)-γ and interleukin (IL)-4 transcripts using the QuantiGene 2.0 Reagent System assay (Panomics, Fremont, CA, USA), according to the manufacturer instructions. Rabbit β-actin (10 ng) was used as a housekeeping gene transcript, to normalise the cytokine quantification. Briefly, rabbit-specific probe sets for IFN-γ (accession number DQ852341), IL-4 (accession number DQ852343) and β-actin (accession number AF309819) were incubated at 55°C with the RNAs from samples at the different bleeding times, in a 96-well mRNA capture plate. After overnight hybridization, the samples were washed three times, supplemented with the pre-amplifier reagent for 1 h at 55°C, and washed again. The amplifier reagent was then added, and the samples incubated for 1 h at 55°C; after further washing, this was replaced by the label probe reagent for 1 h at 50°C. After washing, the chemilumigenic 2.0 substrate was added for 5 min at room temperature, and then the luminescence of each well was read in a luminometer (Modulus™ Microplate Multimode Reader, Turner BioSystems, Sunnyvale, CA). The IFN-γ and IL-4 values are expressed as fold-differences *versus *the baseline calculated from non-stimulated pre-immune RNA of PBMCs, and normalised against their β-actin expression.

### Cytotoxic T-lymphocyte assays

CTL assays are often used to determine the *ex-vivo *specific cytolytic activity of CD8+ T lymphocytes. However, rabbit PBMCs cannot be used as targets in this assay because of their high spontaneous [^51^Cr] release. To overcome this intrinsic difficulty, syngeneic cells were prepared from skin biopsies of each rabbit, as previously described [40], to be used instead of PBMCs, and SV40-immortalised for their possible multiple use during these experiments. The presence of both SV40 viral DNA and RNA transcripts was confirmed by both PCR and RT-PCR in each clone after RNA/DNA extraction from rabbit fibroblasts (data not shown), using the primers V230 (5'-CTT-TGG-AGG-CTT-CTG-GGA-TGC-AAC-T-3') and V231 (5'-GCA-TGA-CTC-AAA-AAA-CTT-AGC-AAT-TCT-G-3').

The target cells were confluent monolayers of SV40-immortalised rabbit autologous skin cells (10^6 ^cells/5-cm Petri dish) infected with 10 PFU/cell of the FP_E6 _or FP_E7 _recombinants. After an overnight incubation and washing in PBS^-^, the cells were dissociated with 0.2% EDTA in PBS^-^, resuspended with 20 ml DMEM10, and pelleted by centrifugation for 5 min at 400 × *g*. The cells were labelled with 50 μCi [^51^Cr] in 100 μl DMEM2 for 2 h at 37°C, washed with 20 ml DMEM10, and soaked in 20 ml DMEM2 for 30 min. The cells were pelleted, resuspended in RPMI with 10% FCS, plated (6 × 10^3^/well) and the effector cells were added.

Autologous effector rabbit PBMCs were used either as freshly prepared or following Ag-stimulation and expansion with IL-2 [40]. These were added to each well at the effector-to-target-cell (E:T) ratios of 30:1 and 15:1. The plates were centrifuged for 5 min at 250 × *g*, and the cells were incubated at 37°C for 4 h. A volume of 50 μl supernatant was transferred from each well into a 96-well LumaPlate containing a solid scintillator (PerkinElmer, Boston, MA). The samples were dried overnight, and the [^51^Cr] release was measured in a MicroBeta JET counter (PerkinElmer). For each sample, the percentage of specific lysis was calculated by dividing the difference between the mean counts per minute of experimental and spontaneous release, by the difference between the mean counts per minute of the total and spontaneous release. For the total [^51^Cr] release, 100 μl 2% Triton X-100 in RPMI was added before harvesting the 50 μl supernatants. All of the assays were performed in triplicate and repeated three to four times for each animal. The cells infected with the FP*env *recombinant [42] were used as an irrelevant negative control.

### Statistical analyses

Statistical analyses were performed using a one-way ANOVA parametric test and Bonferroni/Newman-Keuls analysis of variance using the GraphPad Prism software, version 2.0, as well as the Student t-test. The statistical significance was set as p < 0.05 (*), p < 0.01 (**), p < 0.001 (***).

## Results

### Specific antibody response is higher when CaSki lysates are plated

**Figure 2:**
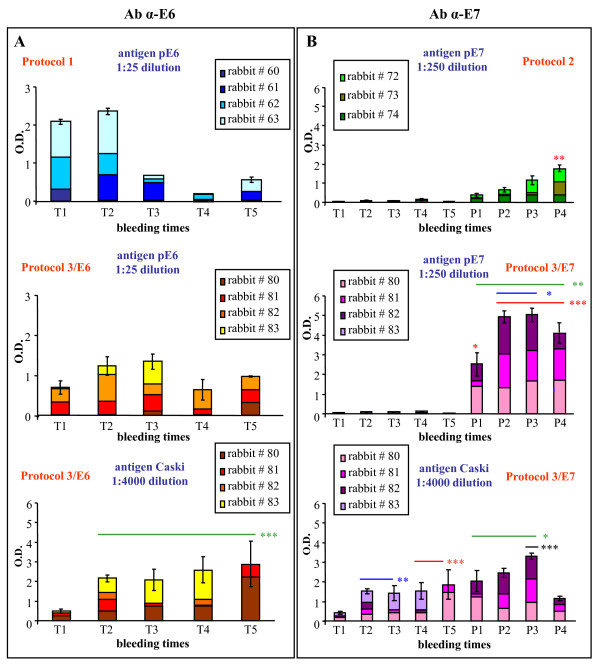
**Analysis of the anti-E6 and anti-E7 humoral responses**. Anti-E6 and anti-E7 antibody titres were determined by ELISA, after plating the E6 (Panel A) or E7 (Panel B) antigens. Heat-inactivated immuno-adsorbed sera were diluted 1:25 or 1:250 for protein-coated plates, and 1:4000 for plates coated with CaSki lysates. The reactions were revealed with goat anti-rabbit HRP-conjugated sera (1:1000) and TMB substrate. The rabbit pre-immune serum from each animal was used as a negative control. Protein boosting with pE7 increased the anti-E7 antibody titres after priming with either FP_E7 _(Protocol 2) or with FP_E6 _+ FP_E7 _(Protocol 3/E7). When CaSki lysates were used, the level of detected antibodies was much higher than after plating the purified proteins with a significant increase of E6 (Protocol 3/E6) and E7 antibodies after priming (Protocol 3/E7) and boosting (Protocol 3/E7). Statistical significances using the ANOVA parametric test are shown: (*) p < 0.05; (**) p < 0.01; (***) p < 0.001.

With the aim of developing a therapeutic vaccine for HPV that can target cells expressing the E6 and E7 oncoproteins, immunised animals were tested for the specific antibody titres. Three groups of rabbits were primed five times with the fowlpox recombinants, and FP_E7 _was followed by three boosts with the corresponding protein (Fig. 1). The humoral response against E6 or E7 was measured in the plasma at different times by ELISA, using plates coated with either HPV-16 pE6 or pE7 proteins or CaSki lysates (Fig. 2). Preimmune serum from each rabbit was used as a negative control. As the rabbits are not syngeneic, results are shown for each single animal to evidence the degree of variability among the animals and the trend shown by each of them overtime. Also, to better compare the E7 humoral response during prime and boost immunisations when the immunogen was delivered either alone (Protocol 2) or together with E6 (Protocol 3/E7), values of the 1:250-diluted E7 sera were plotted on a different scale than the 1:25-diluted E6 sera (Protocol 1 and 3/E6). This does not evidence the similar low response during priming for FP_E6 _and FP_E7_, but clearly shows the enhancement of the response when FP_E7 _is followed by protein boost.

After priming, the rabbits of Protocol 1 and Protocol 3/E6 (Fig. 2A) showed a modest increase of the antibody levels against pE6, similar to that obtained against pE7 before boosting (Fig. 2B, T1-T5), considering the different serum dilution (1:250 *vs*. 1:25). However, after the protein boosting (P1-P4), the increase in the anti-E7 antibody titres was significant (Protocol 2, P4 *vs*. T1-T5, and P4 *vs*. P1; ANOVA parametric test, p < 0.01). In particular, when the rabbits were primed with FP_E6 _+ FP_E7 _(Fig. 2B, Protocol 3/E7), pE7 increased the antibody titres as compared to primary immunisations (P1 *vs*. T1-T5, p < 0.05; P2-P4 *vs*. T1-T5 p < 0.001) and to the previous protein boosting (P2-P3 *vs*. P1, p < 0.05). The response to E7 also increased when the pE7 boosts were preceded by FP_E6 _+ FP_E7 _priming (Fig. 2B, Protocol 3/E7 *vs*. Protocol 2, P1-P4, p < 0.01).

To exclude that sera were able to recognize only the E6 and E7 proteins given as a boosting antigen, plated CaSki lysates were used as a source of native antigen. Overall, the level of antibodies was much higher if compared to the one obtained after plating the corresponding purified proteins. Indeed, a significant increase of E6 (Protocol 3/E6, T2-T5 *vs*. T1, p < 0.001) and E7 antibodies was present after priming (Protocol 3/E7, T2-T3 and T4-T5 *vs*. T1, p < 0.01 and p < 0.001) and boosting (Protocol 3/E7, P3 *vs*. P1, P2, P4, p < 0.001; P1-P3 *vs*. T1-T5, p < 0.05).

### The co-administration of FP_E6 _+ FP_E7 _elicited a balanced Th1/Th2 cytokine response

**Figure 3:**
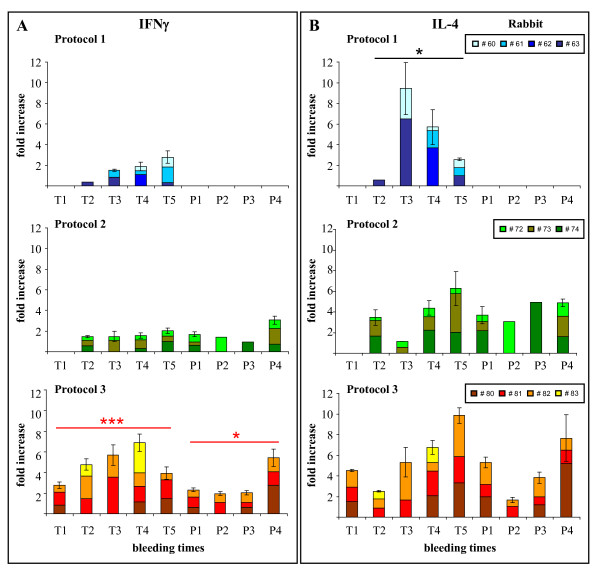
**Th1/Th2 cytokine determination by the QuantiGene 2.0 Reagent system**. The RNAs of the different PBMC samples from all of the bleeding times were used in duplicate to determine the levels of expression of the rabbit IFN-γ (Panel A) and IL-4 (Panel B) transcripts. In all of the animals, the Th2 response was generally higher than Th1 and, in particular, in FP_E6_-immunised animals of Protocol 1 IL-4 production was significantly higher than IFN-γ. IFN-γ production was significantly higher in Protocol 3 than in Protocol 2 both during priming and after the E7 protein boosting. IFN-γ and IL-4 levels are expressed as fold-differences *vs*. baseline, obtained from non-stimulated pre-immune PBMCs, and normalized against β-actin expression. Statistical significances using the Student t-test are shown: (*) p < 0.05; (**) p < 0.01; (***) p < 0.001.

Since the presence of antibodies does not necessarily correlate with cytokine production, we tested the ability of CD4-positive T cells to produce IFN-γ and IL-4 by measuring the specific mRNAs using the QuantiGene 2.0 Reagent System assay. As for ELISA, the results from each single animal were displayed to show the trend of each rabbit overtime, which could be under-evaluated by the degree of variability among non-syngeneic animals. In all of the rabbits, the Th2 response was generally higher than for Th1. In particular, in the FP_E6_-immunised animals, IL-4 production was significantly higher than IFN-γ (Fig. 3B, Protocol 1, Student t-test, p < 0.05). A significant increase in IFN-γ production was noted when the animals were immunised with FP_E6 _+ FP_E7 _(Fig. 3A, Protocol 3 *vs*. 2, p < 0.001) and when the E7 protein boost followed the priming with both recombinant viruses (Fig. 3A, Protocol 3 *vs*. 2, p < 0.05). IFN-γ and IL-4 levels are expressed as fold-differences *vs*. baseline, obtained from non-stimulated pre-immune PBMCs, and normalized against β-actin expression.

### Ex-vivo CTL activity was seen in all of the animals

**Figure 4:**
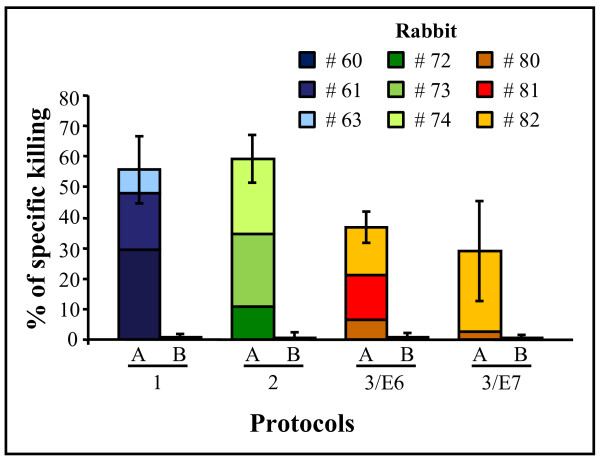
**Functional virus-specific CTL responses**. Effector rabbit PBMCs were used in triplicate, either freshly prepared or following Ag-stimulation and expansion with IL-2. SV40-immortalised autologous target rabbit fibroblasts were labelled with [^51^Cr], and the cytotoxicity determined after the last immunisation. Non-stimulated and FP*env*-stimulated target fibroblasts were used as negative and irrelevant controls. Cytolytic E6- and E7-specific T-cell activity were induced in most of the rabbits (Protocols 1 and 2; [E:T] ratio 30:1). Rabbit # 81 of Protocol 3/E7 was unresponsive to pE7. The results are shown as means of three to four assays.

The cytokine analysis only assesses the type of response of antigen-specific cells, but does not directly demonstrate their cytolytic function. [^51^Cr]-release assays were therefore performed after the last immunisation (Fig. 4). Overall, the results demonstrate that *ex-vivo *CTL activity can be induced in most of the immunised rabbits. Cytolytic T-cells specific for E6 and E7 were detected after Protocol 1 and 2, with a certain variability among the animals, but they did not increase when the rabbits were immunised with both FP_E6 _+ FP_E7 _recombinants or after boosting with the E7 protein (Protocols 3/E6 and 3/E7). Rabbit # 81 was unresponsive to pE7 (Protocol 3/E7). The results are shown as means of three to four assays, which were performed on each animal with either fresh or *in-vitro *expanded PBMCs. Indeed, no significant differences were seen between autologous effector rabbit PBMCs either fresh or Ag-stimulated and expanded with IL-2.

### Challenge with VX2T cells showed tumour regression in all of the animals

**Figure 5:**
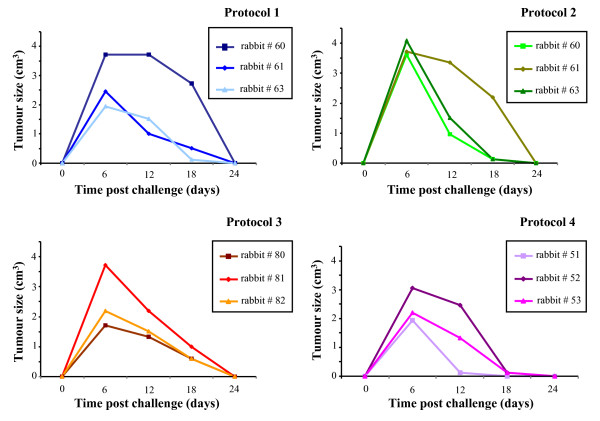
**Tumour cell growth and regression**. Rabbits were injected i.d. with a single dose of non-syngeneic VX2T tumour rabbit cells (10^7 ^cells in 200 μl of PBS^-^), containing the complete genome of CRPV. Tumour size was measured every 6 days with callipers and the volume estimated by the formula width × length × (width + length)/2. The tumour sizes are given for each vaccinated and control (FPwt-injected) animals. All of the rabbits showed a tumour growth up to day 6 post challenge, which was followed by a regression, similar in rabbits vaccinated with the recombinant or FPwt viruses.

*In vitro *propagated VX2T cells, analysed for the expression of the E6 (633 bp) and E7 gene (393 bp) transcripts, were injected at different doses in naïve animals where they developed solid tumours starting from 6 days post-challenge. Tumour size was measured every week with callipers and the volume estimated by the formula width × length × (width + length)/2. All of the animals showed a growing tumour up to day 6 post challenge, but a similar regression was seen thereafter in the rabbits vaccinated either with the FP_E6 _and FP_E7 _recombinants or with the FPwt empty vector (Fig. 5).

## Discussion

Once sexually transmitted, no treatment is available that can eradicate integrated HPV. Over the years, due to the viral strategy of replication, which only occurs in terminally differentiated epithelial cells, HPV vaccine development has been hampered by the difficulty of growing the virus in tissue cultures. VLP-based vaccines targeting the major L1 viral capsid protein of high- and low-risk HPV-types [44] proved effective in preventing persistent infection and precancerous lesions [45]. However, due to the long delay between infection and the appearance of cervical intra-epithelial neoplasias, the long-term durability of the protection by these vaccines has not yet been defined.

Although immunisation with VLPs has the potential to reduce the incidence of cervical cancer [46] and current pharmacological and surgical treatments can reduce or eliminate neoplastic cells, new therapeutic strategies need to be devised for already infected patients [45,47] to prevent or delay disease recurrences. E6 and E7 oncoproteins, which are persistently expressed in HPV-transformed cells [48], represent the main target for immune therapy, as they maintain the proliferative state and prevent apoptosis [49,50].

In the present study, we have described the complete humoral and cellular immune responses that were elicited in three groups of rabbits immunised either with FP_E6 _alone or with FP_E7 _followed by the E7 protein boost. We have demonstrated that: (i) high levels of anti-E6 and anti-E7 antibodies were elicited; (ii) the boosting with the E7 protein increased the humoral response after FP_E7 _priming; (iii) the coadministration of FP_E6 _+ FP_E7 _induced a balanced Th1/Th2 cytokine polarisation; and (iv) a specific CTL response was seen in all of the animals, using autologous fibroblasts as targets.

Many vaccination trials have been performed on patients with cervical cancer, genital warts or papillomas [51,52], using the HPV-16 E6/E7 proteins and DNA or viral vectors, carrying E6/E7 oncogenes but, in spite of the immune response, the already compromised immune system in these subjects often hampered the expected efficacy. The use of viral vectors in a prime-boost regimen has already been shown to enhance the effectiveness of vaccination and a high antibody level was seen to be inversely correlated with disease progression [53,54]. In this study, the antibody response detectable when either the E6 or E7 proteins were plated was very low and did not increase overtime, especially during priming. Co-administration of FP_E6 _+ FP_E7 _did not elicit a synergic effect, but the anti-E7 response was significantly higher when FP_E6 _+ FP_E7 _priming was followed by the pE7 boosting. Since no sequence homology exists between the E6 and E7 proteins, we can hypothesise a non-specific immune stimulation by the doubling of the amount of the FP vector used in Protocol 3. However, when plates were coated with CaSki lysates instead of the E6 or E7 proteins the antibody titre was much higher, which suggests the recognition of conformational epitopes on native CaSki proteins. Conversely, when plates were coated with denatured E7, the high antibody level elicited only after boosts can be ascribed to the recognition of epitopes displayed by the same non-native protein used for immunisation.

Cytokine induction was mainly of the Th2 type, both after the FP_E6 _and the FP_E7 _immunisations. The reduction of the antiviral cellular IFN-γ response has been described for vaccinia and other poxviruses [55] that express genes mimicking the IFN-γ receptor, but was not found in FP-immunised rabbits [56]. In the present study, IFN-γ showed a limited increase after both FP_E6 _or FP_E7 _immunisations, but a significant-one after priming with both recombinants and the E7 protein boost which, given the inability of FP to replicate in mammals, was probably due to the double amount of fowlpox immunogens in Protocol 3 and might explain the balanced Th1/Th2 response.

By eliciting CTLs against HPV-positive tumour cells, therapeutic vaccination represents the most promising treatment to reduce viral load and tumour growth *in vivo *[17]. Many techniques can evaluate cellular immunity, such as cytokine determinations by ELISpot, intracellular staining, and microarrays, none of which are available for the rabbit. As the conventional CTL assay is hampered in these animals by the high spontaneous [^51^Cr]- release by the PBMCs used as targets, we overcame these intrinsic difficulties by using SV40-immortalized syngeneic skin cells as targets and fresh PBMCs or expanded Ag-specific CTLs as effector cells. CTLs were induced in all of the rabbits, but the *ex-vivo *cytolytic activity specific for E6 and E7 did not increase when the animals were immunised with FP_E6 _+ FP_E7 _recombinants, nor after the E7 protein boost. We demonstrated, however, that the rabbit model can be used to verify the presence of cellular immune responses by using autologous fibroblasts. No significant difference was seen between freshly prepared or expanded PBMCs.

Immunisation with VV recombinants elicits a strong immune response and has proven to be well tolerated in animal and human trials. When expressing the E6 or E7 oncogenes, these recombinants have caused tumour regression in patients with advanced cervical cancer and the induction of CTLs specifically directed against infected cells [18,22]. However, the use of VVs for smallpox vaccination causes lytic infection, ulcerations, and scab formation, so that FP recombinants may represent alternative safer immunogens due to their natural host-range restriction to avian species [31,57], their correct expression of transgenes in mammalian cells, and their ability to elicit a complete immune response in vaccinated hosts [58].

Although previously published data described VX2T cells tumorigenicity in New Zealand White rabbits [39], after VX2T cells challenge we observed a complete regression of the solid tumours not only in the rabbits immunised with FP_E6 _and FP_E7_, but also in the animals injected with FPwt. This can be explained by a failure in the system, which, by using non-syngeneic VX2T cells, may have triggered a complete regression as a consequence of the different MHC-I expressed by the host *vs*. the challenging cells.

## Conclusion

The use of conformational epitopes, which can be recognized only after plating CaSki cells, can significantly increase the detectable antibody levels in the immunised rabbits. FP_E6 _and FP_E7 _recombinants might induce CTLs capable of destroying tumour cells and might represent appropriate vectors to elicit anti-tumour immune responses in humans. Further improvements of the recombinants, using the E6 and E7 transgenes deleted of the p53 and p105Rb cellular binding domain, might further increase the safety of the vaccine. Recently, a p53 degradation-defective F47R mutant of HPV-16 E6 was identified, which can restore the function of the p53 protein in HeLa cells [59] and can suppress their proliferation. Similarly, a genetically mutated non-transforming E7 gene (E7GGG), which cannot bind to its p105Rb cellular substrate, could replace the oncogenic E7 counterpart in new constructs and inhibit the E7-expressing TC-1 cell tumour growth in mice [60]. These E6 and E7 genes, genetically modified and inserted into FPwt vectors, will be evaluated for safety, immunogenicity and efficacy for specific elimination of HPV-positive tumour cells.

## Competing interests

The authors declare that they have no competing interests.

## Authors' contributions

AR performed CTL assays, assisted animal immunisations, analysed the data, interpreted the study results, and prepared the manuscript; EP performed animal immunisations, CTL assays, tumour cell cultures, and production of recombinant proteins; SP performed ELISA assays, statistical analyses, assisted animal experiments, and production of recombinant proteins; CZ performed cytokine quantification, analysed the data and the study results and prepared all the figures; CDGM conceptualized, designed, and supervised the whole study. All authors read and approved the final manuscript.
